# Comparative Sequence Analysis of Multidrug-Resistant IncA/C Plasmids from *Salmonella enterica*

**DOI:** 10.3389/fmicb.2017.01459

**Published:** 2017-08-07

**Authors:** Maria Hoffmann, James B. Pettengill, Narjol Gonzalez-Escalona, John Miller, Sherry L. Ayers, Shaohua Zhao, Marc W. Allard, Patrick F. McDermott, Eric W. Brown, Steven R. Monday

**Affiliations:** ^1^Division of Microbiology, Office of Regulatory Science, Center for Food Safety and Nutrition, U.S. Food and Drug Administration College Park, MD, United States; ^2^Division of Public Health Informatics and Analytics, Office of Food Defense, Communication and Emergency Response, Center for Food Safety and Nutrition, U.S. Food and Drug Administration College Park, MD, United States; ^3^U.S. Department of Energy, Oak Ridge Institute for Science and Education Oak Ridge, TN, United States; ^4^Division of Animal and Food Microbiology, Office of Research, Center for Veterinary Medicine, U.S. Food and Drug Administration Laurel, MD, United States

**Keywords:** antimicrobial resistance, IncA/C plasmid, *Salmonella enterica*, next generation sequencing

## Abstract

Determinants of multidrug resistance (MDR) are often encoded on mobile elements, such as plasmids, transposons, and integrons, which have the potential to transfer among foodborne pathogens, as well as to other virulent pathogens, increasing the threats these traits pose to human and veterinary health. Our understanding of MDR among *Salmonella* has been limited by the lack of closed plasmid genomes for comparisons across resistance phenotypes, due to difficulties in effectively separating the DNA of these high-molecular weight, low-copy-number plasmids from chromosomal DNA. To resolve this problem, we demonstrate an efficient protocol for isolating, sequencing and closing IncA/C plasmids from *Salmonella* sp. using single molecule real-time sequencing on a Pacific Biosciences (Pacbio) *RS II* Sequencer. We obtained six *Salmonella enterica* isolates from poultry, representing six different serovars, each exhibiting the MDR-Ampc resistance profile. *Salmonella* plasmids were obtained using a modified mini preparation and transformed with *Escherichia coli* DH10Br. A Qiagen Large-Construct kit™ was used to recover highly concentrated and purified plasmid DNA that was sequenced using PacBio technology. These six closed IncA/C plasmids ranged in size from 104 to 191 kb and shared a stable, conserved backbone containing 98 core genes, with only six differences among those core genes. The plasmids encoded a number of antimicrobial resistance genes, including those for quaternary ammonium compounds and mercury. We then compared our six IncA/C plasmid sequences: first with 14 IncA/C plasmids derived from *S. enterica* available at the National Center for Biotechnology Information (NCBI), and then with an additional 38 IncA/C plasmids derived from different taxa. These comparisons allowed us to build an evolutionary picture of how antimicrobial resistance may be mediated by this common plasmid backbone. Our project provides detailed genetic information about resistance genes in plasmids, advances in plasmid sequencing, and phylogenetic analyses, and important insights about how MDR evolution occurs across diverse serotypes from different animal sources, particularly in agricultural settings where antimicrobial drug use practices vary.

## Introduction

*Salmonella enterica* is one of the most common bacterial causes of foodborne illness, resulting in over 2,100 hospitalizations, 30 deaths, and over 3 billion dollars (US) in direct medical costs each year (Scallan et al., 2011; USDA, 2014). Increasingly, *Salmonella* strains, which can affect both humans, livestock and other animals, are exhibiting multidrug resistance (MDR) to many of the currently available antibiotic therapies, with consequences for consumers, farmers, and public health (Lu et al., 2014). Strains displaying MDR are associated with more severe infections (Varma et al., 2005). In particular, the emergence of MDR strains resistant to both fluoroquinolone and third-generation cephalosporins greatly limits options for treating human salmonellosis (Fair and Tor, 2014), which usually manifests as self-limiting diarrhea, but can progress to life-threatening infections requiring medical intervention (White et al., 2002). The National Antimicrobial Resistance Monitoring System (NARMS) monitors antimicrobial resistance in *Salmonella* isolated from humans, retail meats, and food animals in the United States (FDA, 2016). Some of the MDR *Salmonella* strains identified display resistance to more than 8 antimicrobials. Clinically-relevant antimicrobial resistances among *Salmonella* include the MDR-AmpC phenotype, which is resistant to ampicillin, chloramphenicol, streptomycin, sulfonamide, tetracycline (ACSSuT), as well as to amoxicillin/clavulanic acid, cefoxitin, ceftiofur, and ceftriaxone (Gilbert et al., 2007). While actions have been taken to reduce the use of antibiotics in animal husbandry, variations in use patterns are likely to result in variations in the persistence of MDR phenotypes in different geographic regions.

Although, the composition of bacterial genomes can change rapidly and dramatically in response to antibiotic exposure, the most important changes in the genomes Salmonella and many other genera occur via horizontal gene transfer. Determinants of MDR are often encoded on mobile elements, such as plasmids, transposons, and integrons, which can be transferred from foodborne pathogens to more virulent human pathogens (Winokur et al., 2000; Fricke et al., 2011). This process has greatly contributed to the rapid dissemination of antimicrobial resistance among multiple bacterial genera of human and veterinary importance (Fernandez-Alarcon et al., 2011).

Ideally, we could use comparative analyses of MDR plasmids to understand how resistance evolves among diverse serotypes from different animal sources, particularly in agricultural settings where variations in antimicrobial drug use could exert different selective pressures on *Salmonella* strains. For this reason, complete plasmid sequence information is critical for understanding the nature of antibiotic resistance. The presence of single genes (such as can be detected by PCR) or transferable phenotypes (identified by conjugation experiments) does not provide the detailed genetic context needed to understand the molecular mechanisms involved in bacterial evolution at the allelic level. The same allele might be present in a plasmid as part of a transposon or as an independent coding sequence flanked by degenerate recombination machinery. Interpreting the evolution of antibiotic resistance will differ according to the molecular pathways deduced from the DNA sequence.

However, our understanding about the evolution of MDR is hampered by having only a few closed plasmids available for analysis. Plasmid DNA is often difficult to separate from chromosomal DNA contigs in next generation sequencing (NGS) analyses, particularly if a plasmid is only present in low copy numbers. Furthermore, plasmid sequences frequently contain multiple repetitive regions, which can be difficult to assemble properly from short reads.

One means of resolving that challenge is to use third-generation, single molecule, real-time DNA sequencing on the Pacific Biosciences (PacBio) *RS* II Sequencer, which can provide high-quality reads that are longer than those from any other sequencing platform available as of the beginning of 2017. The use of very long reads greatly facilitates distinguishing plasmid sequences from chromosomal DNA. However, the PacBio technology requires sufficiently high concentrations of high-molecular-weight, low copy number, naturally-occurring plasmids, which has been difficult to achieve using most commercially available isolation kits. Therefore, our project was to develop an efficient plasmid isolation protocol for *Salmonella enterica* serovars that could yield the necessary amounts of high quality plasmid DNA, specifically of large plasmids with a low copy number, which are known to carry genes conferring resistance to a myriad of antibiotics and several toxic heavy metals. To evaluate this method, we prepared plasmid DNA from six different *Salmonella* serovars and analyzed the resulting sequences including (1) 14 additional IncA/C plasmids derived from *S. enterica* and (2) 38 IncA/C additional plasmids derived from different taxa to provide an evolutionary picture of antimicrobial resistance development in IncA/C plasmids.

By analyzing the sequences of these highly similar IncA/C plasmids, we may better understand how antimicrobial resistance is shaped by regional selection pressures, host species, and patterns of antibiotic use, which may provide important insights that could improve future traceback investigations.

## Materials and methods

### Bacterial strains, growth conditions, and antimicrobial susceptibility testing

Six different *S. enterica* isolates, collected from poultry and belonging to serovars Newport, Typhimurium, Infantis, Agona, Kentucky, and Heidelberg, were used in this study (Table 1). These isolates were cultured on trypticase soy agar (TSA; Becton, Dickinson, NJ, USA) and in trypticase soy broth (TSB; Becton, Dickinson, NJ, USA) overnight at 37°C, after which the isolates were stored in TSB containing 20% glycerol at −80°C. All isolates were serotyped by conventional methods, including the standardized NARMS method of *in vitro* antimicrobial susceptibility-testing which uses a panel of 15 antimicrobial agents (amikacin, ampicillin, amoxicillin-clavulanic acid, cefoxitin, ceftiofur, ceftriaxone, chloramphenicol, ciprofloxacin, gentamicin, kanamycin, nalidixic acid, streptomycin, sulfisoxazole, tetracycline, and trimethoprim-sulfamethoxazole), and the TREK Sensititre® automated antimicrobial susceptibility system (Thermo Fisher Scientific, Waltham, MA). Results were interpreted using clinical breakpoints previously established by the Clinical and Laboratory Standards Institute (CLSI).

**Table 1:** List of the metadata for the strains used in this study.

**Accession #**	**Plasmid name**	**Species**	**Location**	**Source**	**Year**
**IncA/C PLASMIDS SEQUENCED IN THIS STUDY**					
CP009409	pCFSAN000405	*Salmonella* Heidelberg	USA_NM	ground turkey	2004
CP009410	pCFSAN007405	*Salmonella* Typhimurium var. O 5	USA_CA	ground turkey	2003
CP009411	pCFSAN007425	*Salmonella* Newport	USA_MD	ground turkey	2002
CP009412	pCFSAN007426	*Salmonella* Agona	USA_CO	ground turkey	2008
CP009413	pCFSAN007427	*Salmonella* Infantis	USA_NM	ground turkey	2009
CP009414	pCFSAN007428	*Salmonella* Kentucky	USA_NM	chicken breast	2006
**IncA/C PLASMID SEQUENCES OBTAINED FROM NCBI**					
CP009560	pCVM22425	*Salmonella* Newport	USA_AZ	cattle	2003
CP009562	pCVM22513	*Salmonella* Newport	USA_NC	cattle	2003
CP009563	pCVM21538	*Salmonella* Newport	USA_GA	chicken breast	n/a
CP009564	pCVM21550	*Salmonella* Newport	USA_TX	swine	n/a
CP009567	pCFSAN000934	*Salmonella* Newport	USA_AZ	canine	2003
CP009570	pCFSAN000941	*Salmonella* Newport	USA_GA	ground beef	2003
FJ621587	pAM04528	*Salmonella* Newport	USA_KS	human	1998
CP000604	pSN254	*Salmonella* Newport	USA_MN	human	2000
JN983043	pSH111_166	*Salmonella* Heidelberg	USA_OH	cattle	2001
JN983045	psH163_135	*Salmonella* Heidelberg	USA_OH	swine	2002
JN983048	psH696_135	*Salmonella* Heidelberg	USA	turkey	2000
JF267651	pSD_174	*Salmonella* Dublin	USA_WI	cattle	n/a
KF056330	p1643_10	*Salmonella* Kentucky	Polen	n/a	n/a
KM670336	pSRC119-A/C	*Salmonella* Senftenberg	Australia	n/a	2000
AP012208	pNDM-1_Dok01	*Escherichia coli*	Japan	human	2009
HQ023861	pPG010208	*Escherichia coli*	Chile	cattle	2004
HQ023862	pUMNK88	*Escherichia coli*	USA_MN	swine	2007
HQ023863	pAPEC1990_61	*Escherichia coli*	USA	turkey	1995
HQ023864	pAR060302	*Escherichia coli*	USA_IL	cattle	2002
JF503991	pNDM10505	*Escherichia coli*	Canada	human	2010
JF714412	pNDM102337	*Escherichia coli*	Canada	human	2010
FJ621586	peH4H	*Escherichia coli*	USA_WA	cattle	2002
KF152885	pSCEC2	*Escherichia coli*	China	swine	2010
CP003225	pKPHS3	*Klebsiella pneumoniae*	China	human	2011
JN157804	pNDM-KN	*Klebsiella pneumoniae*	Kenya	human	2009
JN861072	pNDM10469	*Klebsiella pneumoniae*	Canada	human	2010
JQ010984	pR55	*Klebsiella pneumoniae*	France	human	1969
JX442976	pIncA/C-LS6	*Klebsiella pneumoniae*	Italy	human	2011
KF976462	pRMH760	*Klebsiella pneumoniae*	Australia	human	1997
FJ705807	pRA1	*Aeromonas hydrophila*	Japan	fish	1971
JX141473	pR148	*Aeromonas hydrophila*	Thailand	fish	2008
AB277723	pP99-018	*Photobacterium damselae*	Japan	fish	1999
AB277724	pP91278	*Photobacterium damselae*	Japan	fish	1991
CP000602	pYR1	*Yersinia ruckeri*	USA	fish	1996
CP000603	pIP1202	*Yersinia pestis*	Madagascar	human	1995
CP007636	2012EL-2176	*Vibrio cholerae*	Haiti	human	2012
JN687470	pMR0211	*Providencia stuartii*	Afghanistan	human	2011
FN667743	pXNC1	*Xenorhabdus nematophila*	USA	nematode	1965

For comparative analysis, in addition to the six *S. enterica* plasmids sequenced in this study, we included sequence data from 14 IncA/C plasmids derived from *S. enterica* and 24 IncA/C plasmids derived from different taxa (*Escherichia coli, Klebsiella pneumonia, Aeromonas hydrophila, Photobacterium damselae, Yersinia ruckeri, Yersinia pestis, Vibrio cholera, Providencia stuartii, and Xenorhabdus nematophila*) available at the National Center for Biotechnology Information (NCBI); these are described in Table 1.

### Plasmid isolation

Plasmids were isolated from 5 mL overnight cultures of each strain, grown at 37°C in Luria Bertani (LB) broth containing the appropriate antibiotics at a concentration that assured plasmid maintenance. Bacterial cells were pelleted by centrifugation at 6,000 g/10:00 min/4°C. The resulting bacterial pellet was resuspended in 350 μL resuspension buffer (1X PBS, 40 mM Tris (pH8), 1 mM EDTA) and 200 μg RNaseA was added. This mixture was transferred to 2 mL microfuge tubes, 350 μL lysis buffer (0.2 M NaOH, 1% SDS) was added, and each tube was mixed by inversion several times. After 2 min of incubation at room temperature, 500 μL of neutralization buffer (1.32 M potassium acetate) were added; the mixtures were incubated at room temperature for another 5 min, then centrifuged (20,800 g/10:00 min/4°C). Each supernatant was transferred to a 2 mL microfuge tube and extracted one time using an equal volume of a phenol:chloroform:isoamyl alcohol solution (25:24:1) and tube inversion until the preparation was a homogenous milky color in appearance. Each preparation was centrifuged (20,800 g/8:00 min/4°C) and the aqueous phase transferred to a new 2 mL microfuge tube, avoiding precipitate band present at the interface. The aqueous phase was then centrifuged (20,800 g/10:00 min/4°C) to pellet any residual white precipitate present, then the supernatant (~750 μL) transferred to a 1.5 mL microfuge tube. Next, 0.1 volume (80 μL) of 3M NaOAc (pH5.5) was added and mixed prior to adding 500 μL isopropanol (room temperature), and mixing by inversion (~40X). The solution was centrifuged, as above, to collect the DNA precipitate and washed once with 70% ethanol (room temperature). This last supernatant was discarded and the dried DNA resuspended in 12 μL dIH_2_O. The quantity and quality of this DNA was determined by electrophoresis of a 2 μL aliquot on a 0.6% TBE agarose gel at 120 V (constant).

### Plasmid transformation

We used 40 μL phage-resistant *E. coli* (*E*. coli) DH10B-T1 [Invitrogen catalog number 12033-015. F-*mcr*A Δ(*mrr-hsd*RMS-*mcr*BC) f80*lac*Z ΔM15 Δ*lac*X74 *rec*A1 *end*A1 *ara*D139 Δ(*ara, leu*)7697 *gal*U *gal*K λ—*rps*L *nup*G *ton*A] as host cells for transformation. Transformation was done by electroporation with 1.5–2.0 μL plasmid preparation using large construct parameters, 1.3 kV, 100 Ω, and 25 μFd in a 0.1 cm electroporation cuvette. Typically, time constants ranged from 2.3 to 2.5 ms. Cells were rescued in 750 μL SOC broth at 37°C for 1 h prior to plating on LB agar containing 20 μg/mL chloramphenicol (MP Biomedicals catalog number 02190321, OH, USA) or 100 μg/mL ampicillin (Sigma-Aldrich catalog number A9518, MO, USA). DH10Br-T1 cells containing the desired *Salmonella* antibiotic-resistance plasmids were processed using the Qiagen® Large Plasmid Construct kit (Qiagen catalog number 12462, CA, USA), according to manufacturer's protocol, to obtain suitable concentrations of sequence-quality plasmids. At the completion of each plasmid isolation protocol, DNA purity, and quantity were determined by electrophoresis of (2 μL) each sample on a 0.6% TBE agarose gel at 120 V constant.

### Genome sequencing, assembly, and annotation

We then sequenced these six plasmids using third-generation, single molecule, real-time DNA sequencing on the PacBio *RS II* Sequencer, as previously described (Hoffmann et al., 2013, 2014). Six different 10-kb libraries were prepared following the PacBio sample preparation methods. Each library was sequenced using the C2/P4 chemistry on one single molecule real-time (SMRT) cell with a 120-min collection protocol. The continuous-long-read (CLR) data was assembled *de novo* using the PacBio hierarchical genome assembly process (HGAP2.0). The assemblies outputs from HGAP contain overlapping regions at the end, which can be identified using dot plots in Gepard (Krumsiek et al., 2007). Afterwards, the improved consensus sequences were uploaded in SMRT Analysis, and final consensus and accuracy scores were determined using the Quiver consensus algorithm (Chin et al., 2013). All assembled plasmids were annotated using the NCBI's Prokaryotic Genomes Automatic Annotation Pipeline (PGAAP; Klimke et al., 2009).

### Ideogram of six plasmids

**Figure 1:**
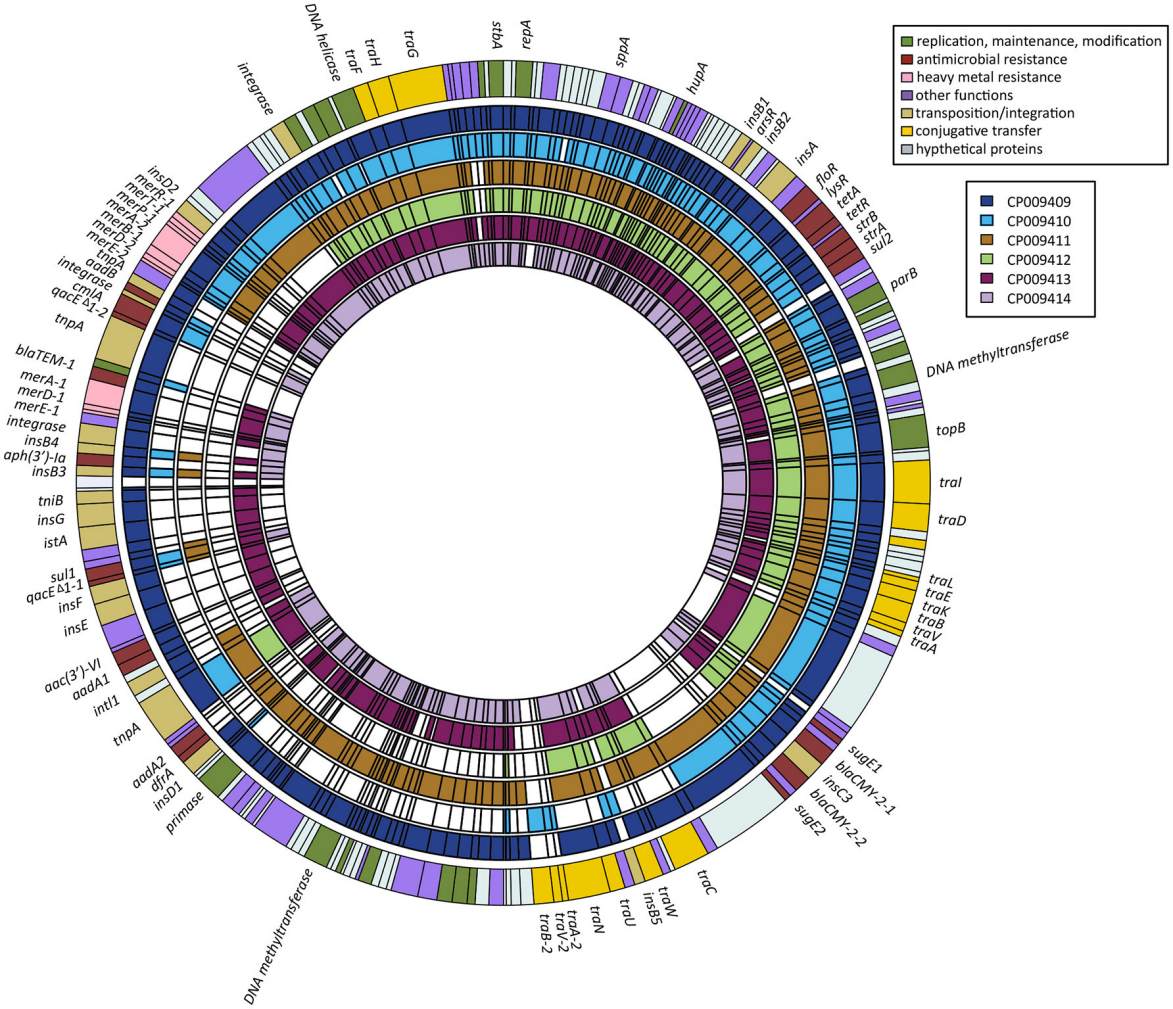
Ideogram showing positions and presence-absence of genes between the six IncA/C plasmids of *S*. *enterica*. The order of the genes in the composite outer ring was modeled after CP009409. Genes not present in that plasmid were added to the composite next to genes they would border in their natural context.

The ideogram (Figure 1) was drawn based on the presence and absence of genes among our six IncA/C plasmids of *S*. *enterica*. The genes for the composite outer ring and each individual plasmid were put into BED format as separate files using a custom Python 2.7 script. We used CP009409 as a template for the composite outer ring, preferentially placing the genes found in other plasmids at the same location as the homolog present in CP009409. Genes were only added to the outer ring if their homolog was absent from CP009409. When it was necessary to add genes to the composite, they were added bordering a gene matching the one they resided next to in their native context. Data from this composite and each plasmid were then loaded into R in BED format as separate files. The ideogram was drawn using Circlize17 with some additional scripting in R to refine the positions of the labels.

### Heat map representation of antimicrobial resistance genes

**Figure 2:**
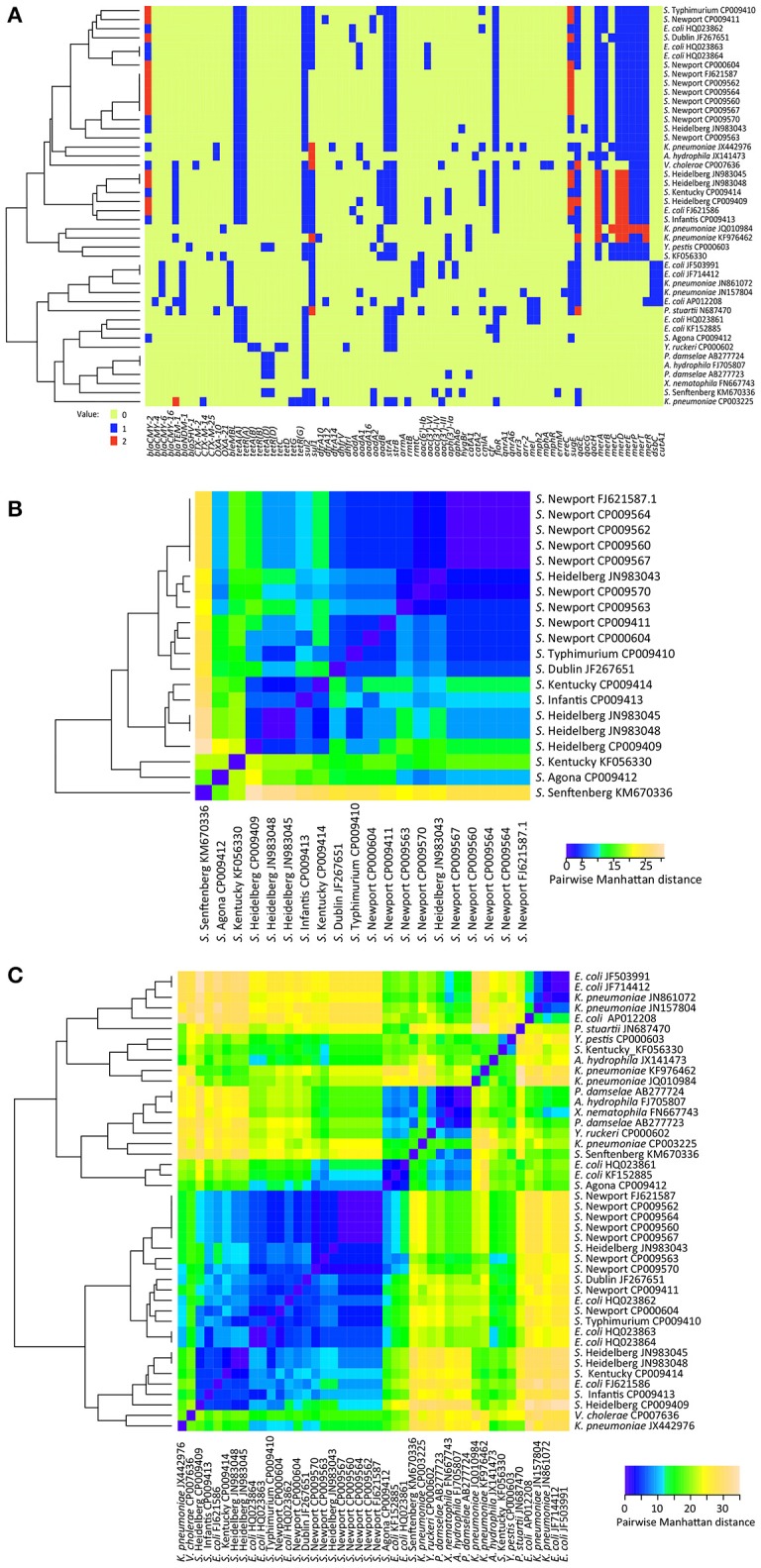
**(A)** Heatmap of presence, absence, and abundance of antimicrobial, metal, and quaternary ammonium compounds resistance genes between 44 IncA/C plasmids. **(B)** Heatmap based on calculating the distance between 20 *Salmonella* IncA/C plasmids in the presence, absence, and abundance of antimicrobial, metal, and quaternary ammonium compounds resistance genes in them. **(C)** Heatmap based on calculating the distance between 44 IncA/C plasmids in the presence, absence, and abundance of antimicrobial, metal, and quaternary ammonium compounds resistance genes in them. The heatmaps were generated using Heatmap.2 in R.

We identified the antimicrobial resistance genes by mapping the closed plasmids to a custom database consisting of 1,379 known resistance genes (Hoffmann et al., 2014). The abundance profile of these genes was then used to construct heatmaps (Figure 2) and clustering using the Heatmap.2 function of the gplots (Warnes et al., 2015) package within R (R Development Core Team, 2015). This enabled us to visualize both the raw counts within each sample and the phenetic relationships among samples, based on Manhattan distances.

### Comparative plasmid sequences analyses

We then performed comparative analyses of the six plasmids with sequence data from 38 complete IncA/C plasmids available at NCBI: 14 extracted from *S. enterica* and 24 isolated from other taxa (Table 1), using the Mauve (Darling et al., 2010) genome alignment v.2 and Geneious R7 v.7.1.3 software packages. The core genes were identified using Ridom SeqSphere+ software v3.0.0 (Ridom GmbH, Münster, Germany). The maximum-likelihood (ML) method of the Genetic Algorithm for Rapid Likelihood Inference (GARLI) software (Zwickl, 2006) was used to construct evolutionary relationships among the closed *Salmonella* plasmid genomes and the wider taxonomic dataset.

We used a reference-free k-mer based approach, embedded in the kSNP v2.1 software(Gardner and Hall, 2013), to identify phylogenetically informative SNP sites (i.e., SNPs shared by two or more strains in the alignment). Briefly, kSNP indexes plasmids into k-mers and then identifies (1) those k-mers that are unique within each plasmid and (2) those k-mers that have a SNP difference in the middle of the k-mer.

### Genbank accession numbers

The plasmid sequences determined in this study have been deposited in the GenBank database under following range of accession numbers: CP009409-CP009414 (Table 1).

## Results and discussion

### Antimicrobial resistance

As shown in Table 2, *in vitro* antimicrobial susceptibility testing results confirmed that our initial set of six *Salmonella* isolates all exhibited the MDR-AmpC resistance profile: resistance to amoxicillin, ampicillin, cefoxitin, ceftriaxone, chloramphenicol, streptomycin, sulfisoxazole, tetracycline, and ceftiofur. Our serovars of *S*. Heidelberg (CFSAN000405), *S*. Infantis (CFSAN007427) and S. Kentucky (CFSAN007428) demonstrated additional resistances to kanamycin and gentamicin, while *S*. Typhimurium (CFSAN007405) and *S*. Newport (CFSAN007425) showed additional resistances to kanamycin, nalidixic acid, and cephalexin, and cephalexin and trimethoprim/sulfamethoxazole, respectively. In the next section we will confirm that the genes enabling these resistance patterns are contained on each respective plasmid.

**Table 2:** List of the resistance genotype-phenotype identified for the six IncA/C plasmids.

	***S*. Kentucky CFSAN007428**	***S*. Newport CFSAN007425**	***S*. Typhimurium CFSAN007405**	***S*. Agona CFSAN007426**	***S*. Heidelberg CFSAN000405**	***S*. Infantis CFSAN007427**
Resistance Phenotype	AMC, AMP, FOX, AXO, CHL, GEN, KAN, STR, FIS, TET, TIO	AMC, AMP, FOX, AXO, CEP, CHL, STR, FIS, TET, COT, TIO	AMC, AMP, FOX, AXO, CEP, CHL, KAN, NAL, STR, FIS, TET, TIO	AMC, AMP, FOX, AXO, CHL, STR, FIS, TET, TIO	AMC, AMP, FOX, AXO, CHL, GEN, KAN, STR, FIS, TET, TIO	AMC, AMP, FOX, AXO, CHL, GEN, KAN, STR, FIS, TET, TIO
Beta-Lactams	bla_cmy−2_, bla_tem−1_	bla_cmy−2_^*^	bla_cmy−2_^*^	bla_cmy−2_	bla_cmy−2_^*^, bla_tem−1_	bla_cmy−2_, bla_tem−1_
Aminoglycosides	*str*A, *str*B, *aadB aph(3)-Ia*	*str*A, *str*B, *aad*A2	*str*A, *str*B, *aad*B	*str*A, *str*B	*str*A, *str*B, *aad*A1, *aad*B, *aac(3')-VI, aph(3')-Ia*	*str*A, *str*B, *aad*A1, *aac(3)-VI*
Tetracyclines	*tetR*(A), *tet*A	*tetR*(A), *tet*A	*tetR*(A), *tet*A	*tetR*(A), *tet*A	*tetR*(A), *tet*A	*tetR*(A), *tet*A
Chloramphenicols	*flo*R, *cml*A	*flo*R	*flo*R, *cml*A	*flo*R	*flo*R, *cml*A	*flo*R
Folate synthesis inhibitors	*sul*1, *sul*2	*sul*1, *sul2, dfra*12	*sul*1, *sul*2	*sul*2	*sul*1, *sul*2	*sul*1, *sul*2
Quaternary ammonium compounds	*quacE*, sugE	*quacE*, sugE^*^	*quacE*, sugE^*^	sugE	quacE^*^, sugE^*^	*quacE*, sugE
Mercury ions	*mer*A^*^, *mer*B, *mer*C, *mer*D^*^, merE^*^, *mer*P, *mer*T, *mer*R	*mer*A, *mer*B, *mer*C, *mer*D, *mer*E, *mer*P, *mer*T, *mer*R	*mer*A, *mer*B, *mer*C, *mer*D, *mer*E, *mer*P, *mer*T, *mer*R		*mer*A^*^, *mer*B, *mer*C, *mer*D^*^, merE^*^, *mer*P, *mer*T, *mer*R	*mer*A^*^, *mer*B, *mer*C, *mer*D^*^, merE^*^, *mer*P, *mer*T, *mer*R

* *Gene is present twice*.

### Plasmid purification and sequencing

All six of the MDR *S. enterica* isolates contained a large IncA/C plasmid, which we identified using replicon typing, as described by Carattoli et al. (2006). We devised an efficient two step protocol for plasmid isolation and extraction: (1) using an osmotically stable resuspension buffer in the initial step to avoid lysing the cells prematurely, a problem observed when using commercially-available buffers, and (2) using *E*. *coli* and commercially-available high molecular weight plasmid isolation kits and their accompanying protocols. The average yield was ~20 μg of high quality DNA.

### Analysis and characterization of the six IncA/C plasmids

All six plasmids were successfully sequenced on the Pacbio *RS II* sequencer, achieving an average coverage between 970X and 3,783X (Table 3). The G+C contents varied from 51.9 to 53.2%. Although, these plasmids differed in size, ranging from 104 in pCFSAN007426 (*S*. Agona) to 191 kb in pCFSAN000405 (*S*. Heidelberg) with between 128 and 226 genes, the genetic organization of each plasmid was very similar (Figure 1). The BLAST analysis of the *repA* gene from these complete sequences confirmed that all six plasmids belonged to the IncA/C incompatibility group. However, in pCFSAN007427 (*S*. Infantis) we identified a large inversion of ~110 kb between the transposase *ins*B2 (locus_tag JV44_00185), which occurs upstream of the *floR* region, and the transposase *ins*B3 (locus_tag JV44_00790), which occurs downstream of the *aad*A region.

**Table 3:** General characteristics of the IncA/C plasmids sequenced in this study.

**Serovar**	**Plasmid name**	**Location**	**Source**	**Year**	**Size**	**#Genes**	**Coverage**	**Accession #**
Heidelberg	pCFSAN000405	USA NM	ground turkey	2004	190,923	226	970X	CP009409
Typhimurium	pCFSAN007405	USA CA	ground turkey	2003	132,146	163	1322X	CP009410
Newport	pCFSAN007425	USA MD	ground turkey	2002	166,496	203	1137X	CP009411
Agona	pCFSAN007426	USA CO	ground turkey	2008	103,586	128	1484X	CP009412
Infantis	pCFSAN007427	USA NM	ground turkey	2009	175,517	217	3783X	CP009413
Kentucky	pCFSAN007428	USA NM	chicken breast	2006	164,924	205	995X	CP009414

We identified 98 genes that were shared across all six IncA/C plasmids; this core plasmid shared 99.9% nucleotide identity and was virtually identical to the plasmid backbone of *S*. Newport SL254, which is very common among MDR isolates from food sources (Welch et al., 2007; Fricke et al., 2009). This backbone includes the replication initiation protein gene, *repA*, and a variety of maintenance genes, such as DNA helicase, DNA binding genes, plasmid stabilization genes, methyltransferase, restriction endonuclease, and the type IV secretion-like conjugative plasmid transfer system (16 genes). Only six of those 98 core genes showed intragenic variability—specifically in the type VI secretion gene, florfenicol/chloramphenicol resistance gene (*flo*R), DNA topoisomerase III (*top*B), DNA binding gene (ner), DNA helicase, and the plasmid stability gene (*stb*A); each variation consisted of a single SNP. Five of those six SNPs were non-synonymous SNPs that would result in changes to the amino acid sequences of the proteins produced (Supplementary Table S1).

Five out of the six IncA/C plasmids contained three type IV conjugative transfer genes regions: (I) *traIDLEKBVA*, (II) *traCWUN*, and (III) *traFHG*. Only pCFSAN007405 (*S*. Typhimurium) did not carry (II) *traCWUN*. Instead, plasmid pCFSAN007405 had an additional region containing a second copy of conjugative transfer genes *tra*A, *tra*V, and *tra*B that were identical to the type IV conjugative transfer genes *tra*A, *tra*V, and *tra*B from region (I) *traIDLEKBVA*, although in reverse order (Figure 1). The main differences among these plasmids appeared to be their accessory regions, most likely a consequence of horizontal gene transfer (Fernandez-Alarcon et al., 2011). These accessory regions tend to be comprised of mobile elements, such as integrons and insertion sequences, carrying transposases, and a variety of resistance genes, which here included genes confering resistance to antimicrobials, quaternary ammonium compounds, and mercury-based antibiotics (Figure 1).

All six plasmids contained the modules *flo*R-*tet*A-*str*AB-*sul*2 and *bla*_CMY−2_-*blc*-*sug*E, and all but pCFSAN007426 (*S*. Agona) also carried the sulfonamide resistance gene, *sul*1 (Table 2). However, certain plasmids contained additional copies of modules or additional genes of interest. Notably, three plasmids, pCFSAN000405 (*S*. Heidelberg), pCFSAN007405 (*S*. Typhimurium), and pCFSAN007425 (*S*. Newport), carried two copies of the *bla*_CMY−2_-*blc*-*sug*E region, confirming previous reports (Welch et al., 2007). Plasmids pCFSAN000405 (*S*. Heidelberg) and pCFSAN007427 (*S*. Infantis) also contained genes for streptomycin resistance, *aad*A1 and gentamycin, *aac*(3′)-VI. The plasmids pCFSAN000405 (*S*. Heidelberg), pCFSAN007405 (*S*. Typhimurium) and pCFSAN007428 (*S*. Kentucky) all also contained an additional streptomycin resistance gene, *aad*B, along with the chloramphenicol resistance gene, *cml*A. The plasmids from pCFSAN000405 *S*. Heidelberg and pCFSAN007428 *S*. Kentucky also carried the kanamycin resistance gene, *aph(3*′*)-*Ia. Furthermore, pCFSAN007425 (*S*. Newport) contains the streptomycin resistance gene, *aad*A2, as well as the trimethoprim resistance gene, *dfra*12. Collectively, the total number of antibiotic resistance determinants in each of these isolates varied between 7 and 14 [*S*. Newport pCFSAN007425 (10), *S*. Typhimurium pCFSAN007405 (10), *S*. Heidelberg pCFSAN000405 (14), *S*. Infantis pCFSAN007427 (11), *S*. Agona pCFSAN007426 (7), and *S*. Kentucky pCFSAN007428 (12)] with all resistance phenotypes correlating positively with the genotypes identified in all six *Salmonella* isolates (Table 2).

In addition to the antibiotic resistance determinants, these plasmids also contained resistance genes protecting against quaternary ammonium and mercury compounds (Figure 1). With the exception of pCFSAN007426, all plasmids contained the quaternary ammonium compound-resistance gene, *qac*E; pCFSAN000405 (*S*. Heidelberg) carried two copies of *qac*E. The same five plasmids carried the *mer*EDBAPTR mercury resistance operon with pCFSAN000405 (*S*. Heidelberg), pCFSAN007428 (*S*. Kentucky), and pCFSAN007427 (*S*. Infantis) carrying two copies of the mercury resistance genes, *mer*A, *mer*D, and *mer*E.

Every resistance gene was surrounded by transposases and/or integrases, suggesting that resistance genes could be transferred easily from one genetic locus to another. Each of our six plasmids contained between 6 and 20 transposition and recombination genes, encoding transposases and integrases; the largest number of these genes were in plasmid pCFSAN000405, which also carried the greatest number of resistance genes.

To complete our comparative analysis of the presence or absence of resistance genes in these plasmids, we constructed heat maps that included data from 38 additional IncA/C plasmids whose sequences were available at NCBI. These heat maps allowed us to generate a color visualization of the data (1) indicating the presence or absence of antimicrobial, metal, and quaternary ammonium compounds resistance genes (Figure 2A), and (2) calculating the distance between IncA/C plasmids in the presence, absence, and abundance of the resistance genes within them (Figures 2B,C). In this analysis, 35 different resistance genes were detected among the 20 *Salmonella* IncA/C plasmids used in this analysis (Figure 2A). Only one resistance gene, *sul2*, was found across all 20 plasmids. Interestingly, two geographically-separated isolates, *S*. Senftenberg KM670336 (Australia) and *S*. Kentucky KF056330 (Poland), showed a distinct resistance gene absence/presence profile when compared to each other, and were also well-separated from the 18 *Salmonella* isolates collected in the USA (Figures 2A,B). The nine *S*. Newport strains isolated in the USA shared similar resistance gene absence/presence profiles (Figures 2A,B) despite coming from different sources (human, swine, cattle, canine, chicken breast, ground turkey, and ground beef). This is especially important, since it demonstrates the stability of the resistance genes within IncA/C plasmids collected from specific geographical locations.

A third heatmap was constructed to establish or determine trends, incorporating the above 20 IncA/C plasmids from *Salmonella* isolates, and adding 24 different IncA/C plasmids from other taxa, available from NCBI at the time of our study. This multi-taxa analysis revealed 76 different resistance genes could be detected among the expanded set of 44 IncA/C plasmids (Figure 2A). This heat map showed that no unique resistance gene profile could be detected for a particular species of bacteria, i.e., there was no cluster within the heat map that included only individual plasmids from the same species (Figures 2A,C). Nonetheless, plasmids isolated from the USA tended to have a similar resistance gene absence/presence profile that could be distinguished from the profiles of plasmids that had been isolated elsewhere in the world. Additionally, the pairwise distance dendrogram demonstrated that plasmids isolated from fish had a similar resistance gene absence/presence profile different from those of plasmids isolated from terrestrial animals that carry more antibiotic resistance genes (Figure 2C). This suggests that each isolate has accumulated resistance genes, which favor survival in its specific environment. Looking at the 14 IncA/C plasmids isolated from humans, 10 of these exhibited a resistance profile similar to the plasmids isolated from fish while four plasmids exhibited a resistance profile more similar to plasmids isolated from land animals.

### Phylogenetic analyses

**Figure 3:**
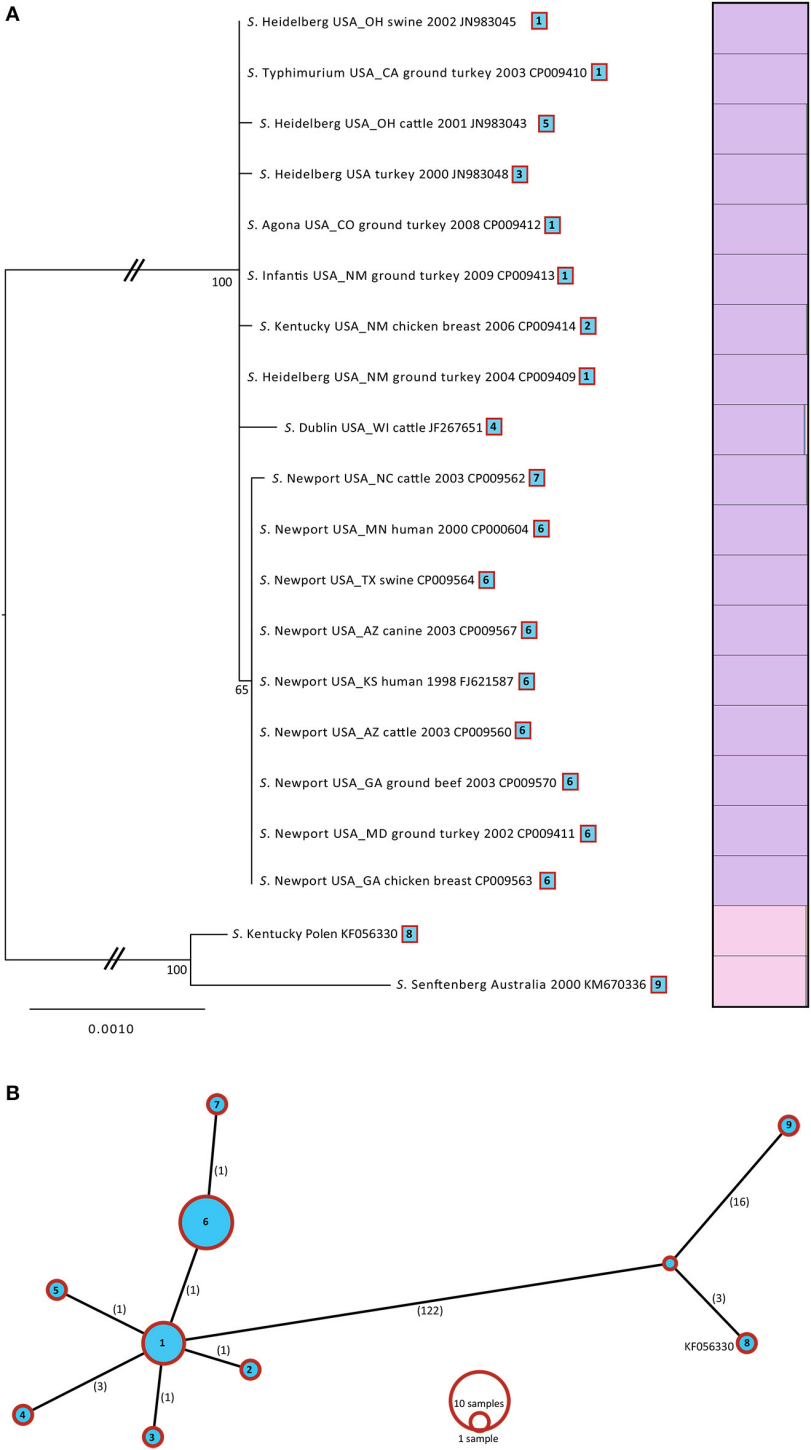
**(A)** ML tree based for 20 IncA/C plasmids isolated from *Salmonella*. A total of 23 genes with 149 variable SNPs were found using Ridom (SeqSphere+). The ML tree was generated in GARLI v.2.0 under the GTR + Γ model of nucleotide evolution and visualized using Figtree v1.3.1. To the right of the tree, a Distruct plots were reconstructed with the gene matrix. The Distruct plot was generated using a model-based Bayesian clustering method implemented in STRUCTURE v2.3.2 and visualized with DISTRUCT v1.1. Different colors represent the different clusters and each bar represents an individual isolate. The fraction of the bar that is a given color represents the coefficient of membership to that cluster (e.g., multicolored bars indicate membership to multiple groups indicative of admixture). The taxa of source for each isolate, geographic location, and date were mapped onto the tree. **(B)** Minimum spanning tree based for 20 IncA/C plasmids isolated from *Salmonella*. The spanning tree was generated in Beast. The numbers of SNPs are shown on each branch.

By comparing the genomes of the six plasmids sequenced in this study with those from publically-available (NCBI) IncA/C plasmids derived from *Salmonella* (Table 1), we identified 67 core genes (Supplementary Table S2). Of these core genes, 44 were 100% identical and 23 had variations in their sequences. We concatenated (total length 14,076 bp), and aligned those 23 variable core genes in order to construct a Maximum Likelihood (ML) tree. The gene matrix consisted of 149 SNP positions 123 of which were informative. The resulting ML tree partitioned the 20 *Salmonella* IncA/C plasmids into two distinct lineages, separated by a mean distance of 132 SNPS, with 100% bootstrap support (Figure 3A).

Lineage I included 18 plasmids having an intra-SNP diversity of 2 SNPs, while Lineage II included only two plasmids, one derived from *S*. Kentucky and the other from *S*. Senftenberg, having a much broader intra-SNP diversity of 19 SNPs. The IncA/C plasmids from Lineage I all belonged to sequence type (ST) 1 A/C_2_ plasmids (Harmer and Hall 2015) and had all been obtained in the USA. Isolates from Lineage II all belonged to ST2 A/C_2_ plasmids (Harmer and Hall, 2015). Based on earlier observations that all of the isolates from a shared geographical area tend to cluster, it seems likely that the plasmids would most likely tend to cluster by geographical location rather than by source or bacterial taxa. The majority of the plasmids from Lineage I could be further divided into two sublineages (65% bootstrap support). Sublineage (SL IA) consisted of *S*. Typhimurium, *S*. Kentucky, *S*. Dublin, *S*. Agona, *S*. Dublin and the isolates of *S*. Heidelberg that had been isolated from chicken breast (3), ground turkey, cattle, and pigs with an intra- diversity of only 1.3 SNPs. Sublinage (SL IIA) consisted of all nine *S*. Newport IncA/C plasmids that had been isolated from different sources, with an intra-SNP diversity of only 0.2.

Although the 23 variable core genes identified within the 20 plasmids had 123 informative SNPs, the haplotype diversity was fairly low, consisting of only 9 haplotypes (Figure 3B). Consequently, there were only a few unique sequences among the core genes from IncA/C *Salmonella* plasmids that were congruent with our tree topology. To provide another way of identifying the number of lineages into which our set of plasmids could be segregated, we used the program STRUCTURE (Falush et al., 2003) to construct groups and then visualized the results using DISTRUCT (Rosenberg, 2004; Figure 3A). Those results (Figure 3A) were consistent with the lineages established by our phylogenetic analyses: two distinct groups, each represented by a different color.

**Figure 4:**
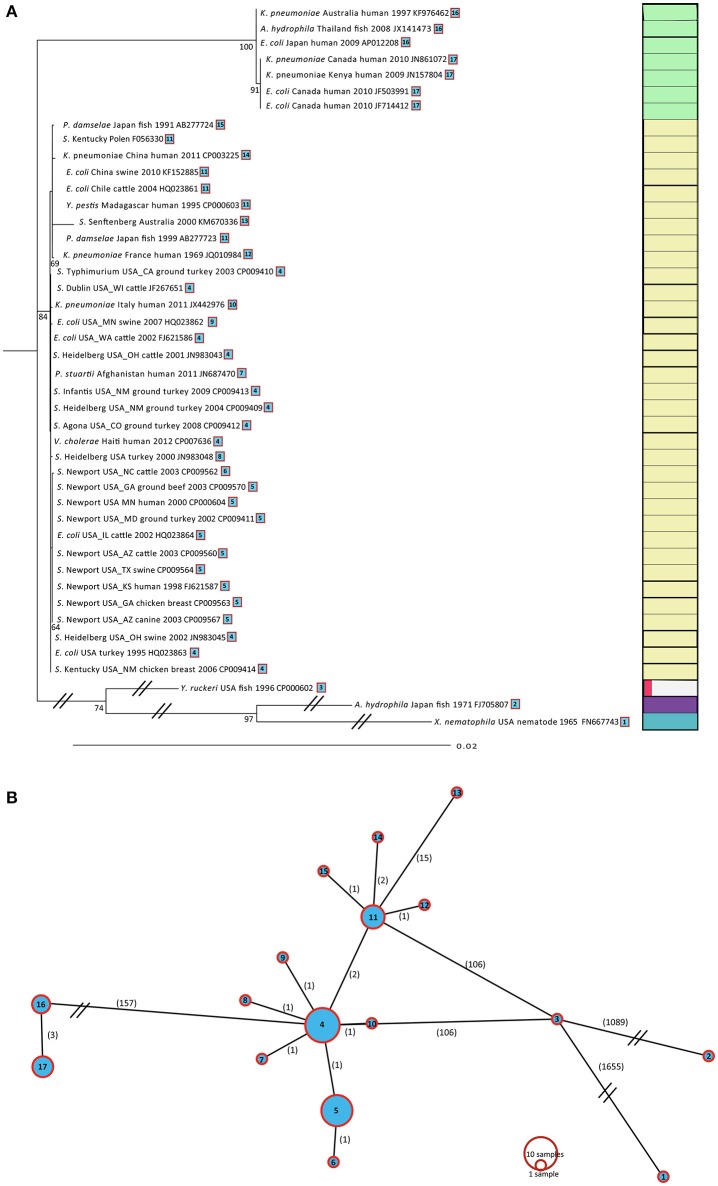
**(A)** ML tree based for 44 IncA/C plasmids. A total of 22 genes with 2,373 variable SNPs were found using Ridom (SeqSphere+). The ML tree was generated as described in Figure 3A. **(B)** Minimum spanning tree based for 44 IncA/C plasmids. The spanning tree was generated in Beast. The numbers of SNPs are shown on each branch.

To provide more precise picture of the evolution and diversity among IncA/C plasmids, we repeated the above analysis, this time including 38 additional IncA/C plasmids from 10 different taxa. Among these 44 IncA/C plasmids a total of 22 core genes could be identified, all containing SNPs (Supplementary Table S3). Those 22 core genes were concatenated (total length 13,299 bp), aligned, and used to create another ML tree (Figure 4A). This gene matrix consisted of 2,366 variable SNP positions, 480 of which were informative. The resulting ML tree partitioned the 44 IncA/C plasmids into three distinct lineages. For this set, Lineage I contained 7 type 2 A/C plasmids, representing three different species (*K. pneumonia, A. hydrophila*, and *E. coli*) isolated and sequenced between 1997 and 2010. Lineage I was divided into 2 sublineages. Sublineage (SL IB) represented plasmids isolated from human and tilapia sources obtained in Australia, Thailand, and Japan; Sublineage (SL IIB) represented four plasmids isolated from humans, obtained in Canada. Lineage II included 34 plasmids, representing eight different species (*Klebsiella pneumoniae, A. hydrophila, Salmonella, P. damselae, Y. pestis, P. stuartii, Vibrio cholerae*, and *E. coli*), isolated between 1991 and 2011. Lineage II could be further divided into three sublineages. Sublineage (SL IC) included nine isolates sequenced type 2 A/C_2_ plasmids from different taxa, isolated from fish and humans, obtained in China, Japan, Madagascar, France and Australia between 1969 and 2011. Sublineage (SL IIC) was comprised of 15 sequenced type 1 A/C_2_ plasmids isolated from human, meat, and animals, obtained mainly from the USA. Sublineage (SL IIIC) was comprised of 10 plasmids from different sources in the USA between 1995 and 2003 and one *E. coli* plasmid isolated from a cow in the USA in 2002. The most diverse lineage, Lineage III, was the most distant from the others; it contained three plasmids obtained from *Y. ruckeri, A. hydrophila*, and *X. nematophila*, isolated from fish (*Y. ruckeri, A. hydrophila)* and nematode (*X. nematophila*) sources. These three lineages were separated by two thousand SNPs; surprisingly, Lineages I and II had an intra-mean diversity of only two SNPs, even though those plasmids had been isolated from very different species at different locations and different times.

Among the complete set of 44 IncA/C plasmids, the 22 variable core genes contained 2,366 informative SNPs. Despite this, the haplotype diversity is fairly low, revealing only 12 haplotypes (Figure 4B), which demonstrated that were fewer unique core gene sequences than the number of IncA/C plasmids obtained across all taxa in our analyses. Using kSNP, we constructed a SNP matrix for each plasmid to further investigate the evolutionary relationships among the closed *Salmonella* plasmid genomes and the wider taxonomic dataset. In contrast to the core-genome approach, we used the full SNP matrix produced by kSNP, which allows for missing data in the matrix. The ML trees produced in this way provided even finer resolution within the Lineages described above (Supplementary Figure S1).

## Conclusions

Although newer sequencing technologies have made it more affordable to perform WGS on bacteria, separating large plasmids from chromosomal contigs is still challenging, particularly if the plasmid is only present low copy numbers. Previous methods of high-molecular-weight plasmid isolation, such as cesium chloride density gradient centrifugation and Solid-Phase Reversible Immobilization (SPRI), involve special equipment and are often very time consuming, especially for a high-throughput and a high-yield approach. This becomes particularly important when one considers that a minimum of 5 μg DNA is required for sequence reads that will extend beyond the size of the repeat domains commonly observed on these plasmids. The problem becomes further exaggerated when the plasmids are to be isolated from *Salmonella* serovars, which have been found to be unusually recalcitrant to those procedures typically used to facilitate such isolations, often yielding little, if any, suitable plasmid DNA. This report presents a plasmid isolation protocol for use with *Salmonella* that is very efficient (yield > 20 μg high quality DNA), fast, and inexpensive.

This method allowed us to isolate and sequence six IncA/C plasmids from six different poultry-derived MDR *Salmonella enterica* serovars. In all six plasmids, we identified genes encoding proteins that correspond to antimicrobial resistance. Each of these antimicrobial-resistance phenotypes could be accounted for by the presence of corresponding resistance genes in these plasmids. There were no instances of resistance that occurred in the absence of a corresponding gene, which demonstrates the importance of plasmid-mediated resistance in *Salmonella*. Our results confirm earlier studies indicating that genetic prediction of phenotypic resistance is a reliable method for resistance monitoring (Hoffmann et al., 2014; Tyson et al., 2015; Zhao et al., 2015). This is especially important as clinical diagnostics move toward culture-independent technologies.

Our study has also provided results that allow a better understanding of the Inc A/C plasmid diversity and evolution in not only *Salmonella* but also across other taxa. The core genome of all the plasmids across the taxa analyzed in this study has a highly conserved backbone of 22 genes. Even though these plasmids were isolated from different bacterial species and from different sources, plasmids isolated from organisms of similar geographic origin tended to have low mutation rates in their core genes. Our phylogenetic tree could distinguish among plasmids obtained from the USA and those plasmids obtained from other parts of the world but did not differentiate among taxa, isolation source, or year. Interestingly, only *S*. Newport genomes clustered tightly together regardless of their source and year of isolation.

Further, differences in SNPs as well as in resistance gene absence/presence profiles between fish-derived plasmids and terrestrial animal-derived plasmids were noticed. Given these patterns, it is likely that local environmental selective pressures are the driving force that gives rise to the diversity among IncA/C plasmids. Although, the majority of the sequence data in our study was acquired from NCBI, and we do not have information regarding the origins of those strains, our analyses suggest that some patients had been infected by strains bearing plasmids with characteristics similar to those from water-borne sources, while other individuals were made ill by strains associated with terrestrial sources. In the future, perhaps resistance gene absence/presence profiles could be used to help guide outbreak investigations of multidrug resistant strains.

## Data availability

GenBank accession numbers for all new sequences are listed in Table 1.

## Author contributions

All authors played an integral part of project conception. Each author has read and approved the final version of the manuscript. Specifically, conceived and designed the experiments: MH, SZ, MA, PM, EB, SM. Performed the experiments: MH, SA, SM. Analyzed the data: MH, JP, NG, JM. Wrote the manuscript: MH, SZ, MA, PM, EB, SM.

### Conflict of interest statement

The authors declare that the research was conducted in the absence of any commercial or financial relationships that could be construed as a potential conflict of interest.

## Supplementary material

Supplementary Table S1 List of the six variable core genes identified between the six IncA/C plasmids sequenced in this study.

Supplementary Table S2 List of the 67 core genes identified between all 20 *Salmonella* IncA/C plasmids Ridom (SeqSphere+).

Supplementary Table S3 List of the 22 core genes identified between all 44 taxa IncA/C plasmids Ridom (SeqSphere+).

Supplementary Figure S1 **(A)** ML tree based for 20 IncA/C plasmids isolated from *Salmonella*. **(B)** ML tree based for 44 IncA/C plasmids isolated from different taxa. The SNPs were found based on k-mer analysis using kSNP. ML trees were generated as described in Figure 3.
