# Supplementary figures and images for: Comparative Sequence Analysis of Multidrug-Resistant IncA/C Plasmids from Salmonella enterica
Source: Front Microbiol. 2017 Aug 7;8:1459. doi: 10.3389/fmicb.2017.01459 (PMC5545573; doi:10.3389/fmicb.2017.01459)

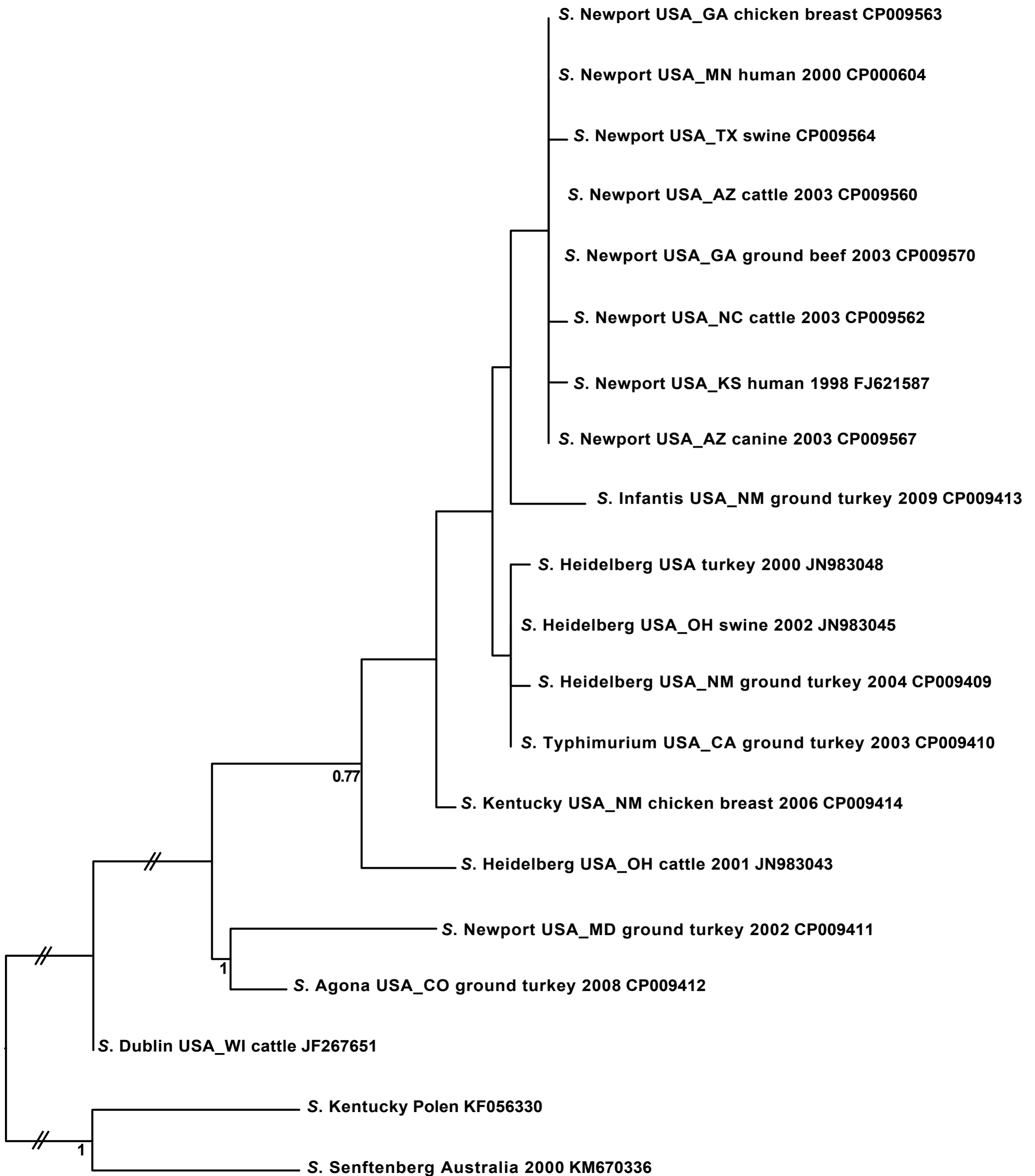

Supplement: Supplementary Figure S1 — (A) ML tree based for 20 IncA/C plasmids isolated from Salmonella. (B) ML tree based for 44 IncA/C plasmids isolated from different taxa. The SNPs were found based on k-mer analysis using kSNP. ML trees were generated as described in Figure 3. [file Image1.PDF]

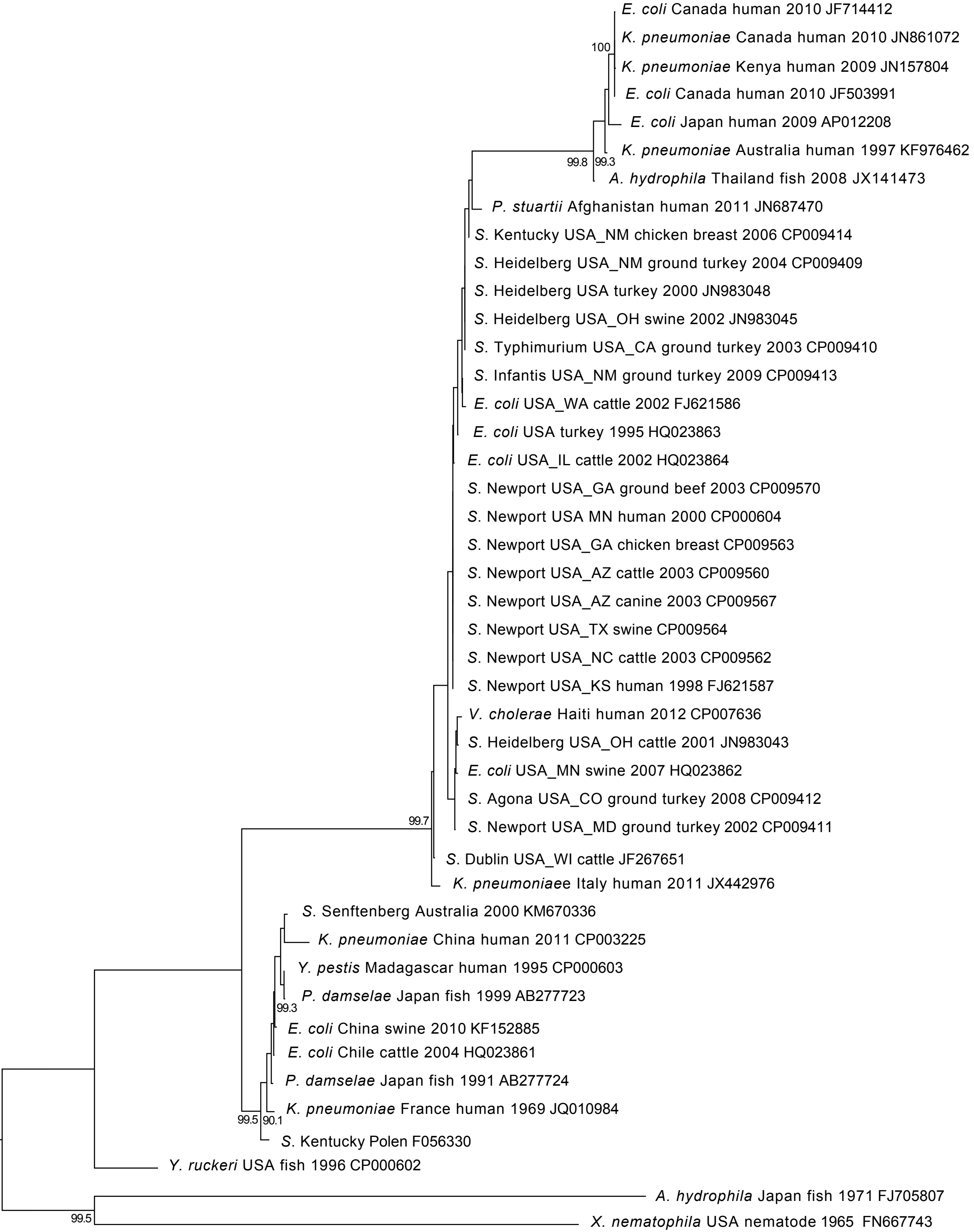

Supplement: Supplementary file 5 [file Image2.PDF]
